# Supplementary material for: Disabled Homolog 2 (DAB2) Protein in Tumor Microenvironment Correlates with Aggressive Phenotype in Human Urothelial Carcinoma of the Bladder
Source: Diagnostics (Basel). 2020 Jan 20;10(1):54. doi: 10.3390/diagnostics10010054 (PMC7168324; doi:10.3390/diagnostics10010054)
Supplement: Supplementary file 1 [file diagnostics-10-00054-s001.pdf]

diagnostics

MDPI

Table S1. Clinicopathological characteristics of 212 patients with bladder cancer undergoing transurethral resection of bladder tumor stratified by DAB2 expression level in stromal area.

		Number of Patients (%)	DAB2 Expression		*P*-value †
			Low	High	
**Total**		**212**	102	110	
Age, years, median (range)		71 (34–94)	69 (34–94)	73 (41–93)	0.19
Gender	Male	181 (86.8)	86	95	0.67
	Female	28 (13.2)	13	15	
clinical T stage	Ta	68 (32.1)	44	24	<0.01
	T1	81 (38.2)	31	50	
	Tis	13 (6.1)	5	8	
	≥T2	50 (23.6)	22	28	
Grade	Low	71 (33.5)	45	26	<0.01
	High	141 (66.5)	57	84	
Carcinoma in situ	Negative	137 (64.6)	77	60	<0.01
	Positive	75 (35.4)	25	50	
Lymphovascular invasion (≥T1)	Negative	65 (49.6)	29	36	0.34
	Positive	66 (50.4)	24	42	
Histological variants	Yes	33 (15.6)	7	26	<0.001
	No	179 (84.4)	95	84	
Lymph node status	N0	207 (97.6)	100	107	0.93
	N1, N2	5 (2.3)	2	3	
Tumor size	≤ 3cm	150 (70.8)	78	72	0.078
	> 3cm	62 (29.2)	24	38	
Number of tumor	single	122 (57.5)	58	64	0.85
	≥ 2	90 (42.5)	44	46	
Infiltration pattern (INF)	a	86 (40.6)	53	34	<0.01
	b	83 (39.2)	37	45	
	c	43 (20.2)	12	31	

† comparing age uging t test and other two groups using the Chi square test.

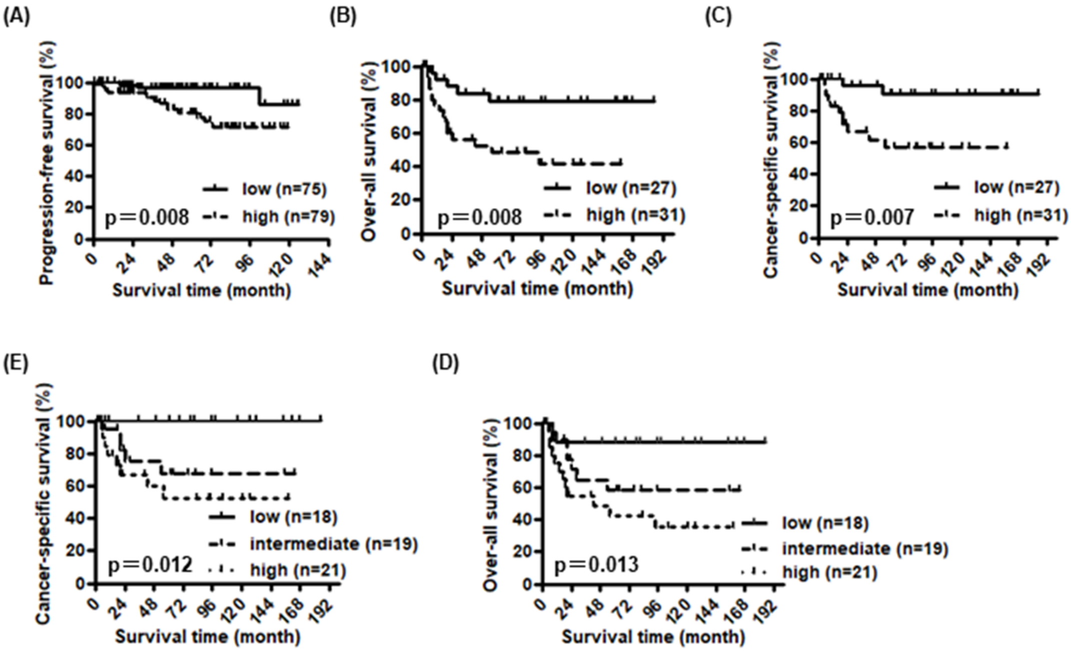

Figure S1. Association between clinicopathological information of the 154 non-muscle invasive bladder cancer (NMIBC) patients and 58 muscle invasive bladder cancer (MIBC) patients and their DAB2 expression in stromal lesions. In NMIBC, there was a significant association between DAB2 expression and progression-free survival (**A**). In MIBC, over-all survival (OS) (**B**) and cancer-specific survival (CSS) (**C**) were significantly lower in patients with high DAB2 expression than that in patients with low DAB2 expression. We stratified the three groups according to expression of DAB2 in cancer and stromal lesions as follows: both low expression; low risk, either high expression; intermediate risk, both high risk; high risk. OS (**D**) and CSS (E) were significantly worse as the risk factors increased.

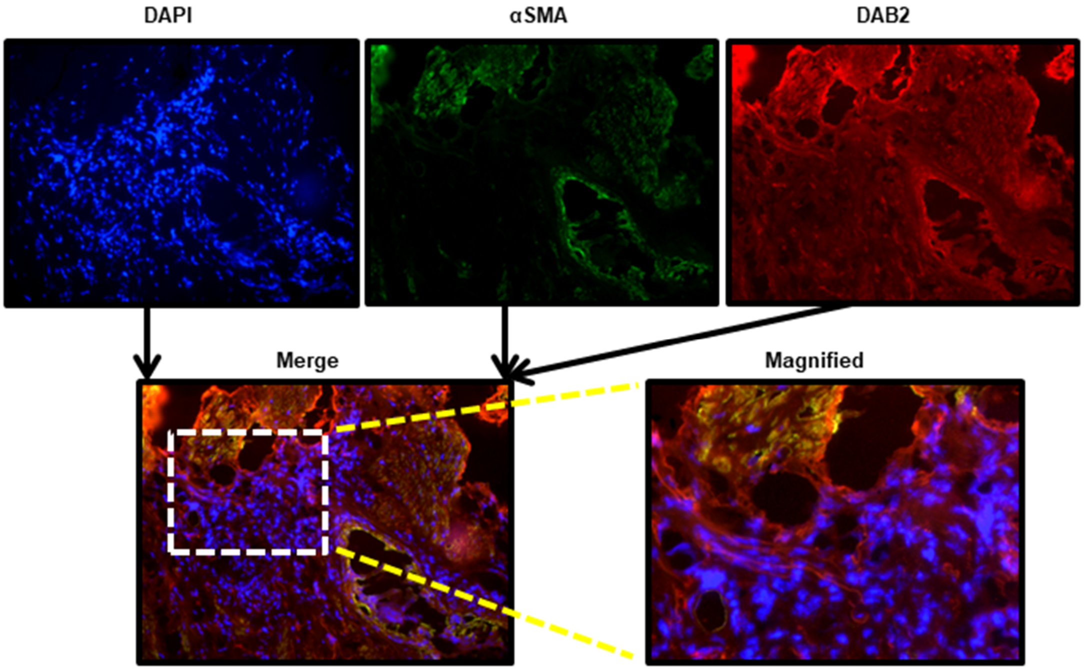

Figure S2. Dual immunofluorescent staining analysis for αSMA (green) and DAB2 (red) illustrates the expression of DAB2 in cancer and stromal tissues in cancerous areas. Overlay images and magnified images are shown (2 panels on the right, below). Original magnification, 200×.

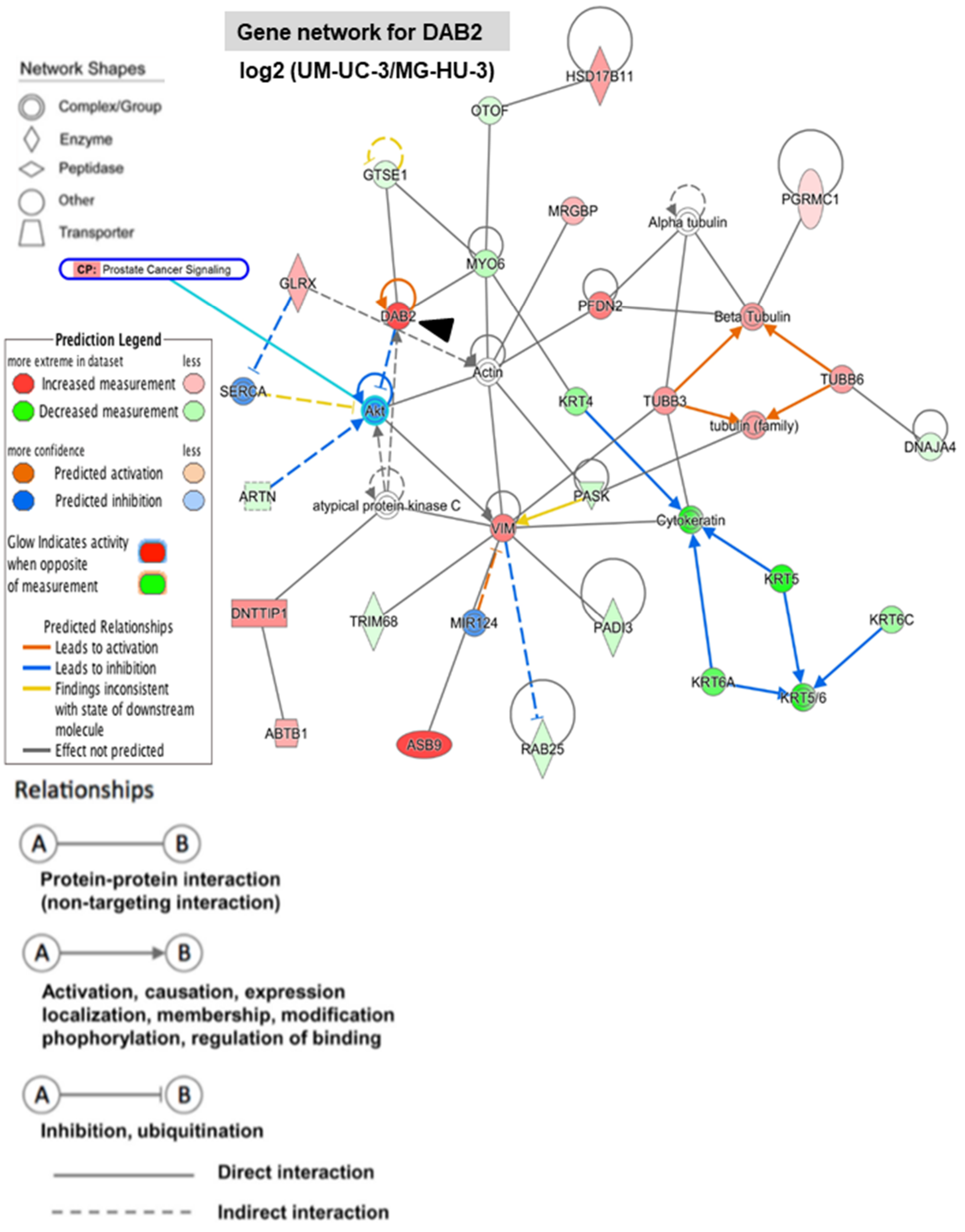

Figure S3. Gene network containing DAB2 generated through ingenuity pathway analysis. We performed gene network analysis as previously described [12]. A total of 279 genes with fold-changes > 2 in all comparisons (UMUC-14 versus MGH-U3, UM-UC-3 versus MGH-U3, and UMUC-3 versus UM-UC-14) were used for molecular network and pathway analysis using Ingenuity Pathway Analysis (IPA, http://www.ingenuity.com) software. Gene networks for *DAB2* and several interaction partners were generated through Ingenuity Pathway Analysis (IPA). Black arrowheads indicate DAB2. Colored nodes are shaded by their relative expression (green: low expression, red: higher expression), with color intensity indicating relative expression. The shape of the node indicates the major function of the protein. Lines denote binding of the products of two genes, while a line with an arrow denotes that one protein acts on another. A dotted line denotes an indirect relationship, and a solid line denotes a direct relationship.
